# Characterization of Chemoresistance in Pancreatic Cancer: A Look at MDR-1 Polymorphisms and Expression in Cancer Cells and Patients

**DOI:** 10.3390/ijms25158515

**Published:** 2024-08-04

**Authors:** Giulia Girolimetti, Barbara Balena, Paola Cordella, Tiziano Verri, Leonardo Henry Eusebi, Maria Pia Bozzetti, Cecilia Bucci, Flora Guerra

**Affiliations:** 1 Department of Experimental Medicine (DiMeS), University of Salento, Via Provinciale Lecce-Monteroni n.165, 73100 Lecce, Italy; giulia.girolimetti@unisalento.it (G.G.); paola.cordella@unisalento.it (P.C.); 2 Department of Biological and Environmental Sciences and Technologies (DiSTeBA), University of Salento, Via Provinciale Lecce-Monteroni n.165, 73100 Lecce, Italy; barbara.balena@unisalento.it (B.B.); tiziano.verri@unisalento.it (T.V.); maria.bozzetti@unisalento.it (M.P.B.); 3 Gastroenterology Unit, IRCCS Azienda Ospedaliero-Universitaria di Bologna, Via Massarenti 9, 40138 Bologna, Italy; leonardo.eusebi@unibo.it; 4 Department of Medical and Surgical Sciences (DIMEC), University of Bologna, Via Massarenti 9, 40138 Bologna, Italy

**Keywords:** drug resistance, pancreatic cancer, transmembrane drug transporter, ABC (MDR/TAP subfamily) transporter

## Abstract

Pancreatic malignancy is the fourth cause of cancer-related death in Western countries and is predicted to become the second leading cause of cancer-related mortality by 2030. The standard therapies (FOLFIRINOX and gemcitabine with nab-paclitaxel) are not resolutive because this type of cancer is also characterized by a high chemoresistance, due in part to the activity of the ATP Binding Cassette (ABC) pumps accounting for the reduction in the intracellular concentration of the drugs. In this work, we analyze the occurrence of single-nucleotide polymorphisms (SNPs) in the MDR-1 gene, in different pancreatic cancer cell lines, and in tissues from pancreatic cancer patients by DNA sequencing, as well as the expression levels of MDR-1 mRNA and protein, by qRT-PCR and Western Blot analysis. We found that gemcitabine-resistant cells, in conjunction with homozygosis of analyzed SNPs, showed high MDR-1 basal levels with further increases after gemcitabine treatment. Nevertheless, we did not observe in the human PDAC samples a correlation between the level of MDR-1 mRNA and protein expression and SNPs. Preliminary, we conclude that in our small cohort, these SNPs cannot be used as molecular markers for predicting the levels of MDR-1 mRNA/protein levels and drug responses in patients with PDAC.

## 1. Introduction

Pancreatic malignancy is the fourth cause of cancer-related death in Western countries and is predicted to become, after lung carcinoma, the second leading cause of cancer-related mortality by 2030 [1]. Late diagnosis represents the main reason due to few preliminary symptoms before the disease reaches an advanced stage [2]. Although in past years there were progresses in the techniques for detecting and managing the disease, pancreatic cancer has still a five-year survival rate of only 9% [3,4].

Pancreatic ductal adenocarcinoma (PDAC) represents 90% of diagnosticated pancreatic neoplasia and the primary risk factors are smoking, alcohol abuse, diabetes mellitus, obesity, aging, family history, and genetic factors [3,5]. The poor prognosis of this malignancy is mainly due to the low rate of early detection, its rapid progression, the development of drug resistance, and the lack of proper therapy. Furthermore, up to now it has not been possible to define specific clinical symptoms, often confused in the first instance with those of other pathologies. In addition, the existing biomarkers, such as CA19-9, have low sensitivity and specificity in the diagnosis of pancreatic cancer [6,7]. Indeed, the main issue of preclinical research is to find circulating biomarkers for the early diagnosis of this type of cancer [8,9]. 

The current standard clinical protocol to care for PDAC is surgery, when the cancer mass is resectable, followed by adjuvant chemotherapy [10]. Nevertheless, only 15% to 20% of patients undergo surgical resection because most of them are diagnosed with unresectable advanced and metastatic disease. To aggravate the clinical picture, there is the characteristic of disease recurrence within a year [11] due to the poor therapeutic results of adjuvant treatment, undetected micrometastasis, and the development of chemoresistance [12]. 

In the last decade, the standard treatment was based on gemcitabine subministration and it has been widely utilized as a first-line drug for advanced pancreatic cancer [13]. Still, since 2011 the gold standard for treating metastatic pancreatic cancer has been represented by two combination regimens: 5-fluorouracil (5-FU)/leucovorin with irinotecan and oxaliplatin (FOLFIRINOX) [14] and gemcitabine with nab-paclitaxel [15,16,17]. A recent study showed that adjuvant therapy with a modified FOLFIRINOX (oxaliplatin, irinotecan, leucovorin, and fluorouracil) regimen resulted in better overall survival (OS) and disease-free survival (DFS) compared to gemcitabine [18].

Nevertheless, the standard therapies are often not resolutive because PDAC is characterized by a high chemoresistance, due in part to the high activity of ATP Binding Cassette (ABC) pumps accounting for the reduction in the intracellular concentration of the drugs [19]. The overexpression of ABC transporters leads to an increased drug efflux, thereby reducing intracellular drug levels and causing drug resistance. Of the 49 human ABC transporters, 15 are implicated in conferring resistance to chemotherapeutic agents in various types of cancers, and one of the most intensively characterized members is the Multidrug Resistance 1 protein (MDR-1, P-glycoprotein, or ABCB1) [20]. As it occurs in different epithelia, the *MDR-1* gene (also called *ABCB1*) is expressed on the apical membrane of epithelial cells in the pancreatic ducts, and it plays a key role in the transport of various molecules across the membrane. For this reason, P-glycoprotein, encoded by the *ABCB1* gene, may be involved in multidrug resistance phenomena (MDR) in various types of cancer [21,22].

Polymorphisms in ABC transporters have been intensively investigated and linked with a varied expression of efflux pumps in different tissue compartments, altered drug levels, and host susceptibilities to several diseases. The *MDR-1* gene can exhibit different genetic variations that can influence the correct function of the protein [23]. In particular, this gene can present various single-nucleotide polymorphisms (SNPs) involved in synonymous or missense mutations [24].

For pancreatic cancer, it was reported that the SNP MDR-1 2677T>G was correlated with drug response in patients receiving adjuvant chemotherapy with gemcitabine [25]. In addition, the wild-type cell lines with the wild-type haplotype MDR-1 2677TT-3435TT were more sensitive to gemcitabine than cells carrying other haplotypes. The MDR-1 2677GG-3435CC haplotypes in these pancreatic cancer cell lines might be associated with other unknown mechanisms that affect the cells’ sensitivity to gemcitabine. Moreover, the abundance of MDR-1 mRNA has also been associated with resistance to gemcitabine [26].

The nonsynonymous mutation in MDR-1 T2677G results in Ala > Ser amino acid changes. This change exhibits lower substrate specificity and reduced drug-stimulated ATPase activity as compared to the wild type [27]. MDR-1 T3435C is a synonymous mutation (Ile1145Ile) even though the variant can alter protein expression by affecting translation efficacy [28]. 

Several trials have been continuously conducted to test the efficacy of different drugs, either alone or in combination with gemcitabine. For instance, a recent study shows the possibility of treating pancreatic cancer with low-dose multiagent therapy with gemcitabine, docetaxel, capecitabine, and cisplatin [29].

Understanding why some patients present severe toxicity, while others do not, could improve the success of chemotherapy; the MDR-1 gene was the first to be evaluated in association with platinum efficacy and/or toxicity [30]. For this purpose, the MDR-1 3435C>T polymorphism influences platinum toxicity; instead, the T allele seems to exert a protective effect on the development of toxicities [30].

Here, we analyze MDR-1 SNP occurrence in pancreatic cell lines and tissues from pancreatic cancer patients. We correlate these polymorphisms with expression levels in cancer cells with different pathological phenotypes, before and after gemcitabine and cisplatin treatment, and in PDAC patient-derived samples in order to understand the role of SNPs in MDR-1 levels and responses to chemotherapy.

## 2. Results

### 2.1. MDR-1 Is Highly Conserved across the Vertebrate Evolutionary Lineage

*MDR-1* emerges as a rather recent gene in animal evolution since (i) it is found in vertebrate genomes only and (ii), across vertebrates, it is restricted to Dipnotetrapodomorpha, which include Dipnomorpha (i.e., lungfishes) and Tetrapoda (i.e., amphibians, reptiles, birds, and mammals). Thus, the emergence of *MDR-1* somehow dates back to vertebrate transition from water to land, i.e., to vertebrate land invasions. 

Human MDR-1 (Figure S1) shares ~70% similarity at the amino acid level when compared to lower Tetrapoda and lungfish orthologs (for details, see Figure S2A), meaning that MDR-1 is highly conserved across the vertebrate evolutionary lineage. It is thus worth noting that of the three polymorphisms discussed in this study (Table 1), (i) rs1128503 (1236T>C) refers to a synonymous mutation that does not result in an amino acid change (G412G), insists on a Gly residue that is invariably conserved across all the species considered, and is located within an evolutionary highly conserved stretch of six amino acids; (ii) rs2032582 (2677T>G) refers to the nonsynonymous mutation that results in a Ser-to-Ala change (S893A), insists on a position where both Ser and Ala residues are found across the species considered, and is located upstream an evolutionarily highly conserved stretch of four amino acids; and (iii) rs1045642 (3435T>C) refers to a synonymous mutation that does not result in an amino acid change (I1145I), insists on an Ile residue that is conserved in all tetrapod species considered, and is located within a not highly conserved stretch of four amino acids (for details, see Figure S2B).

### 2.2. Pancreatic Cancer and Non-Cancer Cell Lines Exhibit Different MDR-1 Polymorphisms 

With the aim of establishing if *MDR-1* polymorphisms may influence the protein expression levels, we first analyzed the DNA from four pancreatic cancer cell lines displaying different pathological phenotypes, from mild to severe: BxPC3, PANC-1, MIA PaCa-2, and YAPC cells. These cells were obtained from the pancreatic duct and are currently used as a model for pancreatic carcinogenesis studies worldwide. Furthermore, we used the normal human pancreatic duct epithelial H6c7 cell line to compare tumor and non-tumor cells. Using the primers listed in Table S1, we analyzed several polymorphisms previously described for other types of cancer, even though their role in cancer is still controversial [31,32]. Among these variations (Table S2), the most common and the most frequently studied SNPs are the two synonymous and one nonsynonymous variant, 1236T>C exon 12, 2677T>G exon 21 [33], and 3435T>C exon 26 [34]. 

Chromatograms from amplicons were analyzed and aligned with the reference sequence NM_001348946.2 (Figure S1) using Nucleotide Blast. Our analysis of the selected polymorphisms in the *MDR-1* gene, whose results are reported in Table 1, revealed that in two of the tested cell lines (non-tumor H6c7 and PANC-1), polymorphisms in exons 12 (1236T>C) and in exon 21 (2677T>G) are present in heterozygosis. In addition, in the YAPC cell line we detected two polymorphisms in exons 21 (2677T>G) and 26 (3435T>C), resulting in homozygosis GG and CC, respectively (Table 1). The BxPC-3 and MIA PaCa-2 cell lines showed no variants. Furthermore, 3435T>C exon 26 polymorphism was present only in the YAPC cell line (1/5, 20% of analyzed cell lines), while 2677T>G exon 21 polymorphism was detected in the H6c7, YAPC, PANC-1 cell lines (3/5, 60% of analyzed cell lines) and 1236T>C exon 12 polymorphism was found in the H6c7 and PANC-1 cell lines (2/4, 50% of analyzed cell lines). We also tested all the other SNPs, listed in Table S2, in all the cell lines and they exhibit a sequence that is the same as the reference one showing no variations. 

### 2.3. The Analysis of MDR-1 Polymorphisms from Patients with Pancreatic Ductal Cancers Reveals a High Frequency of the Selected Variants

To understand whether the frequency of these polymorphisms in PDAC cell lines may be translated in patients, the two most reported variants in the panel of pancreatic cancer cell lines were investigated in a small cohort of individuals diagnosed with pancreatic cancer. Therefore, the analysis for polymorphism 2677T>G in exon 21 and 1236T>C in exon 12 was repeated on DNA extracted from tissues of eight samples (PC1-PC8) of pancreatic cancer patients (Table 2). 

Our analysis of the selected polymorphism in the *MDR-1* gene revealed that in five of the tested patients (PC2, PC3, PC4, PC6, and PC8) 2677T>G is present in heterozygosis (Table 3). Instead, in two patients’ samples (PC5 and PC7) we detected homozygosis for the (2677T>G) polymorphism in exon 21 (Table 3). For the 1236T>C polymorphism in exon 12, patients PC2, PC3, PC4, PC6, and PC8, are heterozygotes, whereas patients PC5 and PC7 are homozygotes for the SNPs. Patient PC1 showed no variation for the selected variants. Thus, both 2677T>G polymorphism in exon 21 and the 1236T>C variant in exon 12 were present in the analyzed samples of 7/8 patients (87.5%), showing a high frequency in the small cohort of analyzed patients.

### 2.4. MDR-1 Expression Is Altered in Pancreatic Cancer Cell Lines by Chemotherapeutic Drug Treatments 

**Figure 1:**
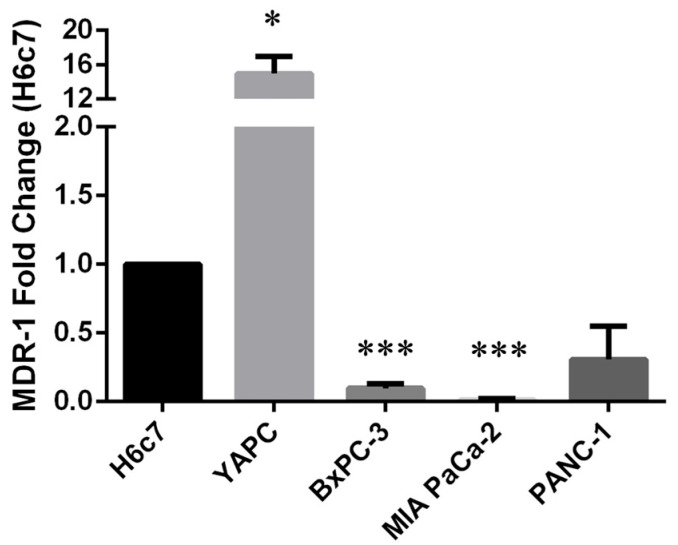
MDR-1 mRNA levels in pancreatic cancer cells. MDR-1 mRNA levels were evaluated in pancreatic cancer cells by qRT-PCR. The fold change was reported compared to H6c7 cells. The relative quantification was considered upregulated when there was a minimum of a two-fold change. Data represent the mean ± SEM of at least three independent experiments. *p < 0.05; *** *p* < 0.001.

In order to study the correlation between the occurrence of polymorphisms and the expression level of *MDR-1*, we decided to perform quantitative Real-Time PCR (qRT-PCR) analysis on pancreatic cancer cells compared to H6c7 pancreatic non-tumoral cells, used as control. As shown in Figure 1, we found high MDR-1 mRNA levels in the YAPC cell line characterized by the presence of homozygous polymorphisms in exons 21 and 26 (2677T>G and 3435T>C, respectively) and very low levels in the other PDAC cell lines, in particular BxPC-3 and MIA PaCa-2, bearing no variation or heterozygous status for the selected variants, compared to the non-tumor cell line.

**Figure 2:**
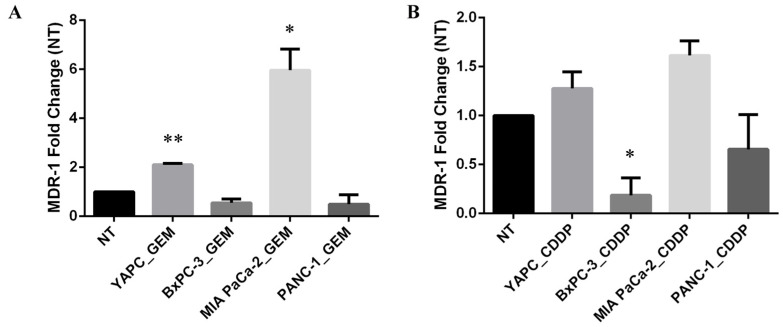
MDR-1 mRNA levels in pancreatic cancer cells. MDR-1 mRNA levels were evaluated in pancreatic cancer cells after 72 h of gemcitabine (GEM) (**A**) or cisplatin (CDDP) (**B**) exposure. The EC50 values, calculated for each drug and each cell line, were used for the treatment. The fold change was reported compared to non-treated (NT) cells. The relative quantification was considered upregulated when there was a minimum of a two-fold change. Data represent the mean ± SEM of at least three independent experiments. * *p* < 0.05; ** *p* < 0.01.

Thus, to investigate whether treatment with gemcitabine or cisplatin might induce alterations in mRNA expression levels of *MDR-1*, we performed qRT-PCR in PDAC cell lines before (non-treated, NT) and after treatment with gemcitabine (GEM) or cisplatin (CDDP) for 72 h (Figure 2). EC50 concentrations calculated for each cell line were used for GEM and CDDP drugs. 

Interestingly, the levels of MDR-1 mRNA were unchanged in BxPC-3 and PANC-1 cell lines upon GEM treatment, while the most GEM-resistant YAPC cells, characterized by the presence of homozygous polymorphisms 2677T>G and 3435T>C in exons 21 and 26, respectively, and the most GEM-sensitive MIA PaCa-2, bearing no variations for the selected SNPs, showed a significant increase in MDR-1 mRNA levels (Figure 2A). In contrast, the levels of MDR-1 were unchanged after the treatment with CDDP in YAPC and MIA PaCa-2 cell lines and were reduced in BxPC-3 and PANC-1 cells (Figure 2B). Therefore, treatment with different chemotherapeutic drugs showed opposite effects on MDR-1 mRNA expression.

Then, we decided to validate the qRT-PCR data, analyzing the levels of MDR-1 protein in cells before and after treatment with GEM and CDDP. For this purpose, we treated four PDAC cell lines using drugs at their respective EC50 concentrations and analyzed protein expression by Western Blotting.

**Figure 3:**
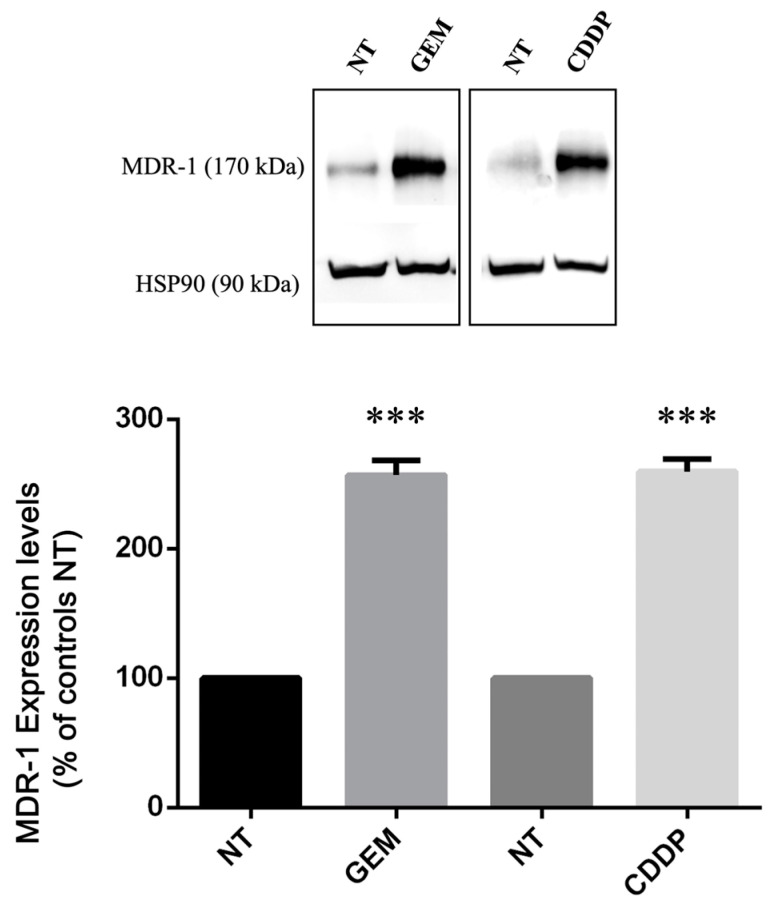
MDR-1 protein levels in YAPC pancreatic cancer cells. Relative protein abundance of MDR-1 was assessed by Western Blot analysis in non-treated (NT) and in gemcitabine (GEM)- and cisplatin (CDDP)-treated cells using antibodies against MDR-1 and HSP90. Histograms show differences in MDR-1 levels obtained after quantification by densitometry normalization against HSP90. Data represent the mean ± SEM of at least three independent experiments. *** *p* < 0.001.

Only YAPC cells, which reported the higher levels of MDR-1 mRNA (Figure 1), showed MDR-1 protein expression (Figure 3), while in the other cell lines, the protein was not detectable both before and after gemcitabine and cisplatin treatments, despite having used three different antibodies and a femtogram-level sensitive chemiluminescence kit.

Interestingly, as shown in Figure 3, we found a significant increase in MDR-1 protein levels in YAPC cells after treatment with GEM and CDDP. Taking into consideration the effect of CDDP in the YAPC cell line (Figure 2B and Figure 3), our data reveal a poor correlation between mRNA and protein abundance for MDR-1.

In light of the different behavior after drug treatments, we performed an in silico analysis using STITCH (http://stitch.embl.de/ accessed on 25 July 2024), a database of protein–chemical interactions that integrates various sources of experimental and manually curated evidence with text-mining information and interaction predictions [35]. Interestingly, we found that in the ABCB1-mediated response to the drugs (GEM and CDDP), it is possible to count other actors that could affect the interaction (Figure S3A,B). Based on these results, further analyses need to be performed to understand whether the different behavior of MDR-1 levels in our cells may be due to interactors of this protein or of the used drug.

### 2.5. Analysis of MDR-1 Expression in Pancreatic Cancer Patients

With the aim to transfer data obtained on PDAC cultured cell lines to human samples, we first performed an in silico analysis using the Human Protein Atlas database (https://www.proteinatlas.org/ accessed on 25 July 2024) [36]. Genome-wide transcriptome obtained from patients diagnosed with PDAC was related to clinical outcomes. In particular, among the analyzed genes, we found that high levels of mRNA in ABCB1 are considered a favorable prognostic marker (Figure S4). Nevertheless, these data refer to primary tumors of selected patient cohorts. Thus, we have no information about ABCB1 SNPs, the effect of chemotherapeutic treatment, and protein level alterations. 

**Figure 4:**
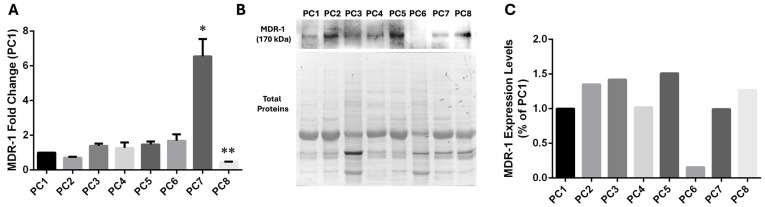
MDR-1 levels in tissues from pancreatic cancer patients. (**A**) MDR-1 mRNA levels were evaluated after RNA extraction from tissues from pancreatic cancer patients. The fold change was reported compared to the PC1 sample, which displayed no polymorphism. The relative quantification was considered upregulated when there was a minimum of a two-fold change. Data represent the mean ± SEM of at least three independent experiments. * *p* < 0.05, ** *p* < 0.01. (**B**,**C**) The MDR-1 protein was evaluated in tissues from pancreatic cancer by Western Blotting normalization on total protein. Different expressions were estimated using PC1 as a control. Data represent one experiment using protein extracts obtained from patient tissues.

For this reason, we then analyzed MDR-1 mRNA levels in tissues from pancreatic cancer patients. Despite the low number of patients analyzed, we found higher MDR-1 mRNA levels in the PC7 sample, which is characterized by homozygous polymorphisms in exons 21 (2677T>G) and 12 (1236T>C). In contrast, levels of MDR-1 mRNA were unchanged in PC5, characterized by the same homozygous polymorphisms (Figure 4A). Then, we decided to validate qPCR data by Western Blot analysis on protein extracts purified from patient tissues to evaluate MDR-1 protein levels (Figure 4B,C). The data reveal a poor correlation between mRNA and protein abundance for MDR-1. Indeed, surprisingly, high levels of MDR-1 mRNA expression in PC7 do not correlate with a high abundance of the MDR-1 protein. Patients with the same homozygous polymorphisms (PC5 and PC7 patients) showed similar MDR-1 protein levels to patients without polymorphisms (PC1) or heterozygous polymorphisms (PC2-4, PC6, PC8). PC5 and PC7 do not show an increase in MDR-1 protein levels, confirming that in our patient’s cohort, the homozygous polymorphisms in MDR-1 are not related to an increase in the protein levels. 

Thus, although the number of patients is minimal, based on our data on patients, MDR-1 SNPs do not affect MDR-1 mRNA and protein levels and cannot be used as molecular markers for predicting drug responses in patients with PDAC. Certainly, these data are very preliminary and they must be confirmed on a larger patient-sample cohort.

## 3. Discussion

In the literature, a great number of studies are focused on the role of *ABCB1* genetics in various diseases, taking into consideration the different phenotypes, such as MDR-1 expression, and protein levels. Nevertheless, the genetic association of *MDR-1* SNPs with expression, activity, drug response, and disease risk is still controversial [37,38]. Here, we considered the three most common SNPs in the protein coding region, rs1128503 (1236T>C, Gly412Gly), rs2032582 (2677T>G, Ser893Ala), and rs1045642 (3435T>C, Ile1145Ile) [39] that have been the target of drug-response and disease association studies with contrasting results [37,38]. Then, we analyzed MDR-1 levels in samples with different statuses of the genetic variants to understand whether the latter may have an effect on mRNA and protein levels. 

In the panel of PDAC cell lines that we included in this study, the only cell line in which we found important variations was YAPC, where we detected two polymorphisms in homozygosis in exons 21 (2677T>G) and 26 (3435T>C). rs1045642 (3435T>C) is the most frequent among the *MDR-1* genetic polymorphisms [34]. The 3435T>C polymorphism is a synonymous SNP, and thus it does not cause a change in the amino acid sequence. Synonymous SNPs should not alter the function of the protein as the amino acid sequence is not altered, but the *MDR-1* 3435T>C polymorphism at codon 1145 (Ile1145Ile) has been demonstrated to cause several changes in mRNA level and expression [40]. rs2032582 (2677T>G) is a nonsynonymous mutation that results in Ala > Ser amino acid changes. It has been found that the *MDR-1* 2677T>G SNP could be associated with multidrug resistance [33]. Analysis of MDR-1 expression revealed high basal levels of mRNA and protein in YAPC cells and a small increase in mRNA levels after treatment with gemcitabine, allowing us to hypothesize a correlation between homozygosis SNPs, MDR-1 expression, and response to gemcitabine. Indeed, among the panel of pancreatic cell lines, YAPC cells are characterized by a gemcitabine chemoresistant phenotype [9]. In addition, in this cell line, MDR-1 protein levels were increased upon both GEM and CDDP treatments. Platinum-based drugs are not moved by MDR-1 but they were reported to upregulate its expression and activity; this is relevant in combination chemotherapy with other drugs such as paclitaxel or gemcitabine, which are transported by this protein [41]. 

In contrast, the most gemcitabine-sensitive MIA PaCa-2 cells displayed a high increase in MDR-1 mRNA levels after treatment probably as a failed attempt of the cells to respond to the drug. Indeed, it was reported in other types of cancers, such as, for instance, melanoma, non-small-cell lung cancer, small-cell lung cancer, epidermoid carcinoma, and ovarian cancer, that after gemcitabine treatment, cells are characterized by an increase in MDR-1 expression together with the acquisition of more sensitivity to gemcitabine compared to parental cell lines. This was demonstrated to be because P-glycoprotein overexpression may cause cellular stress, resulting in increased gemcitabine metabolism and sensitivity [42]. Nevertheless, we could not detect the protein in MIA PaCa-2 cells, both untreated or treated with GEM or CDDP, presumably due to the absence or very low levels of the MDR-1 protein undetectable by Western Blot analysis. 

We also identified two patients with PDAC that exhibit homozygosity for the rs2032582 (2677T>G) SNP in exon 21 and the rs1128503 (1236T>C) polymorphism in exon 12, which was a silent mutation (same amino acid in the protein). As we found high levels of MDR-1 mRNA in one of the two MDR-1 mutated patient samples, we hypothesized a possible correlation between polymorphism in homozygosis and expression levels also in human samples and in response to chemotherapeutic treatment. Instead, when we measured MDR-1 protein levels by Western Blot analysis on cell lysates from patient tissues, PC5 and PC7 did not show an increase in MDR-1 protein levels compared to the other PDAC patients, confirming that in our patient cohort, the homozygous polymorphisms in MDR-1 are not related to an increase in the protein levels. 

Looking together at the data obtained, limited by the low number of patient samples analyzed, we found a poor correlation between mRNA and protein abundance for MDR-1. This could be very important in the future evaluation of the role of this gene in chemoresistance. 

The clinical information regarding patients included in our cohort revealed that all the patients were diagnosed with advanced disease (stage IV) and died within 6 months from diagnosis, except for patients PC3 and PC5 who were diagnosed at stage III and II, respectively. Patient PC5 was diagnosed with early-disease (stage II) PDAC, underwent surgery and treatment with FOLFIRINOX, and today this patient does not have a recurrence of the disease. In contrast, PC7 was diagnosed with advanced disease (stage IV), did not undergo treatment, and died three months after the tissue sample collection. In our small cohort of patients, only PC4 was treated with GEM and PC5 was treated with Folfirinox. Therefore, based on the characteristics of this pathology and patient management, it is impossible to clarify whether MDR-1 mRNA and protein levels increase in patients with homozygous polymorphisms when treated with chemotherapeutic drugs as we showed in the YAPC cell line. The lack of strong genetic association for 1236T>C and 3435T>C may be due to the fact that this is a non-causative variant and the contribution to chemoresistance may depend on other indirect regulatory mechanisms [38].

Taken together, although these SNPs do not appear to play a predominant role in altering MDR-1 levels and, subsequently, in chemoresistance, they may have a role in other causal variants that regulate MDR-1 transcription [43,44]. Our data do not allow for clear conclusions, indicating that more research is needed before a real link between these polymorphisms and drug resistance can be established. Hence, additional studies will be important to confirm these preliminary data and identify the molecular mechanisms underlying the resistance of gemcitabine and platinum-derived therapy and its toxicity.

## 4. Materials and Methods

### 4.1. Case Series and Samples

Tissue samples were collected from 8 pancreatic cancer patients enrolled between 2022 and 2023 within the frame of the EXOPanc (role of exosomes in pancreatic cancer progression) study, approved by the Area Vasta Emilia Centro Ethical Committee. All cases were diagnosed at the S. Orsola-Malpighi University Hospital, Bologna, Italy. The mean (±standard deviation) age of patients was 69.2 (range from 59 to 85 years). All patients were diagnosed with poorly differentiated pancreatic adenocarcinoma G3 (histologic grading system) [45]. Two patients were diagnosed with stage II/III (PC3, PC5), while the majority of cases presented stage IV pancreatic cancer and distant metastases to other areas of the body, such as the liver. The detailed characteristics of the patient cohort are summarized in Table 2. An alpha-numeric code (from PC1 to PC8) was assigned to maintain anonymity. Tissues were collected together with the informed consent obtained in compliance with the Helsinki Declaration. Internal review board protocols were followed for the collection of samples. 

### 4.2. Cell Lines

A set of 4 different human PDAC cell lines were used. MIA PaCa-2 (Cellosaurus Research Resource Identifiers RRID:CVCL_0428 [46]) and PANC-1 (RRID:CVCL_0480 [46]) cells were grown in DMEM (Euroclone, Milano, Italy), while YAPC (RRID:CVCL_1794 [46]) and BxPC-3 (RRID:CVCL_0186 [46]) cells were maintained in RPMI-1640 medium (Euroclone). Media were supplemented with 10% FBS, 2 mM glutamine, 100 U/mL penicillin, and 10 mg/mL streptomycin (Euroclone, Milano, Italy). 

In this study, we decided to use four pancreatic cancer cell lines with different genotypic and phenotypic characteristics to take into consideration. Indeed, individually, these models recapitulate some, but not all, aspects of the tumor progression. Different aspects were evaluated to select the cell lines, such as the genotypic status of commonly altered genes (KRAS, TP53, CDKN2A, and SMAD4) and derivation of the cell line, such as primary tumor, ascites, metastasis, the cell differentiation, and the epithelial or mesenchymal characteristics. BxPC-3 resulted in a cell line with mild pathological phenotypes due to its intrinsic phenotypic and genotypic characteristics. PANC-1, MIA PaCa-2, and YAPC cell lines were reported as mesenchymal-type with a severe pathological phenotype. Furthermore, the latter derived from ascitic fluid, therefore from metastatic cells, suggesting a very severe pathological condition. Indeed, the YAPC cell line was derived from the malignant ascites of a 43-year-old Japanese man with an advanced pancreatic cancer of the tail. MIA PaCa-2 has been established in continuous culture from an undifferentiated human pancreatic adenocarcinoma of a 65-year-old man. PANC-1 was cultured from a primary tumor of a 56-year-old male with adenocarcinoma in the head of the pancreas which invaded the duodenal wall. Poor differentiation and mesenchymal-type cells were reported for all of them. In contrast, BxPC-3 was cultured from a biopsy specimen of a histologically confirmed adenocarcinoma of the body of the pancreas. No evidence of metastasis was reported. Differentiation was reported as moderate to poor, and an epithelial phenotype was described. YAPC, MIA PaCa-2, and PANC-1 cells carried mutations in the pancreatic cancer key gene KRAS, while no variations were reported in BxPC-3 [46,47,48,49,50,51,52,53]. The immortalized epithelial cell line derived from normal human pancreatic duct epithelial cell H6c7 (RRID:CVCL_0P38 [46]) was cultured in Keratinocyte SFM, + 2.5 µg EGF human recombinant + 25 mg bovine pituitary extract (Thermo Fisher, Whaltman, MA, USA, Gibco Cat#: 10724-011 and 37000-015) supplemented with 1× Antibiotic-Antimycotic (Gibco Cat#: 15240-062). All the cell lines were grown in a 5% CO_2_ incubator at 37 °C. MIA PaCa-2 and PANC-1 were kindly provided by Prof. Miriam Martini (University of Turin, Turin, Italy) [54], while YAPC, BxPC-3, and H6c7 were a gift from Dr. Loredana Moro (New York University, New York, NY, USA and National Research Council Bari, Bari, Italy) [55]. 

### 4.3. DNA, RNA, and Protein Extraction from Patient Tissues

Tissues derived from pancreatic cancer biopsies were ground in liquid nitrogen with mortar and pestle to homogenize the sample and subjected to DNA, RNA, and protein purification using an AllPrep DNA/RNA/Protein Mini Kit (Qiagen, Hilden, Germany) according to the manufacturer’s protocols.

Quality control and quantification of the extracted DNA and RNA were performed using a NanoDrop ND-1000 spectrophotometer (Thermo Scientific, Waltham, MA, USA). Protein extracts were quantified using the BCA assay (Thermofisher Scientific, Waltham, MA, USA). 

### 4.4. DNA, RNA, and Protein Extraction from Cell Lines

Whole genomic DNA was extracted from each cell line using a Mammalian Genomic DNA Miniprep Kit (Sigma-Aldrich, St. Louis, MO, USA) according to the manufacturer’s protocols. Total RNA was extracted from PDAC cell lines in the presence or not of gemcitabine and cisplatin treatments using an AurumTM Total RNA Mini Kit (Biorad, Hercules, CA, USA) according to the manufacturer’s instructions. Quality control and quantification of the extracted DNA and RNA were performed using a NanoDrop ND-1000 spectrophotometer (Thermo Scientific, Waltham, MA, USA). For protein purification, cells were lysed in Laemmli Buffer (100 mM Tris–HCl pH 6.8, 4% SDS, 20% glycerol, and 0.2% blue bromophenol) and then quantified using the BCA assay (Thermofisher Scientific, Milano, Italy) or by Stain-free gels (Bio-Rad, Hercules, CA, USA).

### 4.5. Genotyping using the Polymerase Chain Reaction (PCR)

DNA samples were diluted to an optimal concentration of 50 ng/µL and each amplification reaction was performed with 1 µL of genomic DNA in a 50 µL total reaction volume containing 5 µL of 10× DNA-free PCR Buffer (${-MgCl}_{2}$), 1.5 µL of 50 mM DNA-free ${MgCl}_{2}$, 1 µL of 10 mM dNTP mix, 1 µL of each 10 µM primer, and 0.5 µL of 5 U/ µL DNA-free Platinum Taq DNA Polymerase.

PCR was performed in a Bio-Rad T100 Thermal Cycler with a cycle program at 94 °C for 2 min, 35 cycles at 94 °C for 30 s, annealing temperature (Ta) for 30 s, 72 °C for 30 s, and 4 °C for hold. The amplification products were separated on an agarose gel 1× with a final volume of 50 mL and then they were purified with a PureLink^®^ PCR Purification Kit and sequenced to a total volume of 2.2 µL using 1 µL of 6 ng/ µL of amplicon and 1.2 µL of 10 µM forward primer according to the manufacturer’s recommendations. The primers selected for the DNA amplification of selected exons are listed in Table S1. Amplicons, obtained from 3 independent PCR, were sequenced by BMR Genomics. 

In this study, chromatograms from amplicons obtained from sequencing were analyzed and aligned with the reference sequence on Nucleotide Blast to determine the presence of possible polymorphisms. In the results, we reported the variations that were confirmed in all three experiments. The primers used in PCR analysis are listed in Table S1.

### 4.6. Cell Treatment and Viability Measurement in the Presence of Cisplatin

Cell viability for the YAPC and BxPC-3 cell lines was measured by the MTT (3-(4,5-dimethylthiazol-2-yl)-2,5-diphenol tetrazolium bromide) assay. The conversion of MTT by mitochondrial succinate dehydrogenase was used as an indicator for cell density determination as already described [9,56]. Cells were seeded in 96-well plates (Corning, NY, USA) (5 × 10^3^ cells/well) in a complete medium; after 24 h, cells were washed twice in PBS and incubated in different media. To evaluate cytotoxicity, different concentrations of cisplatin (Enzo Life Science, Farmingdale, NY, USA) were used (1 µM, 5 µM, 10 µM, 50 µM, and 100 µM). Plate reading was performed on a Multilabel Plate Reader (Victor X5, PerkinElmer, Waltham, MA, USA) at 570 nm. The half-maximal effective concentration (EC50) value was used as a measure of the drug potency. EC50 was calculated after 72 h as the concentration that resulted in a 50% decrease in the number of cells compared to that of the untreated cells using GraphPad Prism software (Version 6.0, GraphPad, San Diego, CA, USA). Regarding cisplatin and gemcitabine treatments, cells were seeded in complete medium. After 24 h, cells were incubated with drugs for 72 h. The EC50 values (Table 4) were used for the treatment of all PDAC cell lines and subsequent analyses with qPCR and Western Blotting. In particular, we used gemcitabine at 800 nM and 168 nM for YAPC and BXPC as previously determined [9] and 6 µM for both cell lines for cisplatin treatments. 

### 4.7. Quantitative Real-Time PCR (qRT-PCR)

RNA extraction and quantitative Real-Time PCR (qRT-PCR) were performed as described previously [57,58]. Reverse transcription was performed using an iScript cDNA Synthesis kit (Bio-Rad). 

Real-Time PCR analysis was performed on a CFX96 System (Bio-Rad). For the *MDR-1* gene, the following primers were used: Forward 5’-GGGATGGTCAGTGTTGATGGA-3’ and Reverse 5’-GCTATCGTGGTGGCAAACAATA-3’.

Data were normalized to the mRNA level of RPLP0, used as a housekeeping gene [59], and relative mRNA expression was calculated using the ΔΔCt method and expressed as “fold change”. The relative quantification was considered significant when there was a minimum of a two-fold change. The specificity of PCR products was confirmed by melting curve analysis.

### 4.8. Western Blotting

Proteins were separated by SDS-PAGE and subsequently electroblotted onto polyvinylidene fluoride (PVDF) Immobilon-P membranes (Millipore, Billerica, MA, USA) as previously reported [57,58]. Primary antibodies against MDR-1 (sc-13131, Santa Cruz Biotechnology, Santa Cruz, CA, USA) and Hsp90 α/β (sc-13119, Santa Cruz Biotechnology) were incubated overnight. Anti-mouse (#1706516, Biorad, Hercules, CA, USA) secondary antibody was incubated for 1 h at r.t. Images were acquired by a ChemiDoc MP Imaging System and analyzed by Image Lab TM software version 6.0.1 (Bio-Rad Laboratories). Protein expression levels were quantified by densitometry normalization against the housekeeping bands.

### 4.9. Statistical Analysis

Results are represented as mean ± standard error (SEM). Statistical significance was determined for all experiments using Student’s t-test for unpaired data (* *p* ≤ 0.05, ** *p* ≤ 0.01, and *** *p* ≤ 0.001).

## Figures and Tables

**Table 1:** Polymorphisms identified in pancreatic tumor and non-tumor cell lines.

Cell Lines	Exons	Analyzed Polymorphism	Genotype
H6c7	Exon 12	rs1128503 (1236T>C)	Heterozygote T/C
	Exon 21	rs2032582 (2677T>G)	Heterozygote T/G
	Exon 26	rs1045642 (3435T>C)	no variation (T/T wt)
BXPC3	Exon 12	rs1128503 (1236T>C)	Not determined
	Exon 21	rs2032582 (2677T>G)	no variation (T/T wt)
	Exon 26	rs1045642 (3435T>C)	no variation (T/T wt)
YAPC	Exon 12	rs1128503 (1236T>C)	no variation (T/T wt)
	Exon 21	rs2032582 (2677T>G)	Homozygote G/G
	Exon 26	rs1045642 (3435T>C)	Homozygote C/C
PANC-1	Exon 12	rs1128503 (1236T>C)	Heterozygote T/C
	Exon 21	rs2032582 (2677T>G)	Heterozygote T/G
	Exon 26	rs1045642 (3435T>C)	no variation (T/T wt)
MIA PaCa-2	Exon 12	rs1128503 (1236T>C)	no variation (T/T wt)
	Exon 21	rs2032582 (2677T>G)	no variation (T/T wt)
	Exon 26	rs1045642 (3435T>C)	no variation (T/T wt)

**Table 2:** Clinical characteristics of patients diagnosed with pancreatic cancer.

Characteristics	
Gender (F/M)	3/5
Age, mean years (range)	69.9 (59–85)
Size of lesion, median mm (range)	36.9 (15–59)
Tumor stage, n (%)	
II	1 (12.5%)
III	1 (12.5%)
IV	6 (75%)
Lesion location, n (%)	
Pancreatic head	6 (75%)
Pancreatic body	1 (12.5%)
Pancreatic tail	1 (12.5%)
Distant metastases, n (%)	
M0	2 (25%)
M1	6 (75%)

**Table 3:** Polymorphisms identified in tissue from pancreatic patients.

Patients	Exon	Analyzed Polymorphism	Genotype
PC1	Exon 21 Exon 12	rs2032582 (2677T>G) rs1128503 (1236T>C)	No variation (T/T wt) No variation (T/T wt)
PC2	Exon 21 Exon 12	rs2032582 (2677T>G) rs1128503 (1236T>C)	Heterozygote T/G Heterozygote T/C
PC3	Exon 21 Exon 12	rs2032582 (2677T>G) rs1128503 (1236T>C)	Heterozygote T/G Heterozygote T/C
PC4	Exon 21 Exon 12	rs2032582 (2677T>G) rs1128503 (1236T>C)	Heterozygote T/G Heterozygote T/C
PC5	Exon 21 Exon 12	rs2032582 (2677T>G) rs1128503 (1236T>C)	Homozygote G/G Homozygote C/C
PC6	Exon 21 Exon 12	rs2032582 (2677T>G) rs1128503 (1236T>C)	Heterozygote T/G Heterozygote T/C
PC7	Exon 21 Exon 12	rs2032582 (2677T>G) rs1128503 (1236T>C)	Homozygote G/G Homozygote C/C
PC8	Exon 21 Exon 12	rs2032582 (2677T>G) rs1128503 (1236T>C)	Heterozygote T/G Heterozygote T/C

**Table 4:** EC50 values ± SEM for gemcitabine (GEM) and cisplatin (CDDP) in PDAC cell lines.

Cell line	EC50 GEM (nM) [9]	EC50 CDDP (µM)
BxPC-3	168 ± 32.02	6 ± 1.5
YAPC	800 ± 125	6 ± 1.51
PANC-1	187 ± 24.63	12 ± 1.47
MIA PaCa-2	18 ± 1.64	8 ± 1.35

## Data Availability

Datasets are available on request from the authors.

## Supplementary Materials

The following supporting information can be downloaded at https://www.mdpi.com/article/10.3390/ijms25158515/s1.
