# Supplementary material for: Characterization of Chemoresistance in Pancreatic Cancer: A Look at MDR-1 Polymorphisms and Expression in Cancer Cells and Patients
Source: Int J Mol Sci. 2024 Aug 4;25(15):8515. doi: 10.3390/ijms25158515 (PMC11312866; doi:10.3390/ijms25158515)
Supplement: Supplementary file 1 [file ijms-25-08515-s001.zip › ijms-3112486-supplementary.pdf]

International Journal of *Molecular Sciences*

MDPI

Article

Characterization of Chemoresistance in Pancreatic Cancer: A Look at MDR-1 Polymorphisms and Expression in Cancer Cells and Patients

Giulia Girolimetti [1], Barbara Balena [2], Paola Cordella [1], Tiziano Verri [2], Leonardo Henry Eusebi [3,4], Maria Pia Bozzetti [2], Cecilia Bucci [1*] and Flora Guerra [2*]

[1] Department of Experimental Medicine (DiMeS), University of Salento, Via Provinciale Lecce-Monteroni n.165, 73100 Lecce, Italy; paola.cordella@unisalento.it (P.C.); giulia.girolimetti@unisalento.it (G.G.)
[2] Department of Biological and Environmental Sciences and Technologies (DiSTeBA), University of Salento, Via Provinciale Lecce-Monteroni n.165, 73100 Lecce, Italy; barbara.balena@unisalento.it (B.B.); tiziano.verri@unisalento.it (T.V.); maria.bozzetti@unisalento.it (M.P.B.)
[3] Gastroenterology Unit, IRCCS Azienda Ospedaliero-Universitaria di Bologna, Via Massarenti 9, 40138 Bologna, Italy; leonardo.eusebi@unibo.it
[4] Department of Medical and Surgical Sciences (DIMEC), University of Bologna, Via Massarenti 9, 40138 Bologna, Italy

* Correspondence: cecilia.bucci@unisalento.it (C.B.); flora.guerra@unisalento.it (F.G.)

```
-136                                                 a
-135 ttcgagtagcggctcttccaagctcaaagaagcagaggccgctgt
 -90 tcgtttcctttaggtctttccactaaagtcggagtatcttcttcc
 -45 aaaatttcacgtcttggtggccgttccaaggagcgcgaggtcgga

   1 ATGGATCTTGAAGGGGACCGCAATGGAGGAGCAAAGAAGAAGAAC
   1  M  D  L  E  G  D  R  N  G  G  A  K  K  K  N

  46 TTTTTTAAACTGAACAATAAAAGTGAAAAAGATAAGAAGGAAAAG
  16  F  F  K  L  N  N  K  S  E  K  D  K  K  E  K

  91 AAACCAACTGTCAGTGTATTTTCAATGTTTCGCTATTCAAATTGG
  31  K  P  T  V  S  V  F  S  M  F  R  Y  S  N  W

 136 CTTGACAAGTTGTATATGGTGGTGGGAACTTTGGCTGCCATCATC
  46  L  D  K  L  Y  M  V  V  G  T  L  A  A  I  I

 181 CATGGGGCTGGACTTCCTCTCATGATGCTGGTGTTTGGAGAAATG
  61  H  G  A  G  L  P  L  M  M  L  V  F  G  E  M

 226 ACAGATATCTTTGCAAATGCAGGAAATTTAGAAGATCTGATGTCA
  76  T  D  I  F  A  N  A  G  N  L  E  D  L  M  S

 271 AACATCACTAATAGAAGTGATATCAATGATACAGGGTTCTTCATG
  91  N  I  T  N  R  S  D  I  N  D  T  G  F  F  M

 316 AATCTGGAGGAAGACATGACCAGGTATGCCTATTATTACAGTGGA
 106  N  L  E  E  D  M  T  R  Y  A  Y  Y  Y  S  G

 361 ATTGGTGCTGGGGTGCTGGTTGCTGCTTACATTCAGGTTTCATTT
 121  I  G  A  G  V  L  V  A  A  Y  I  Q  V  S  F

 406 TGGTGCCTGGCAGCTGGAAGACAAATACACAAAATTAGAAAACAG
 136  W  C  L  A  A  G  R  Q  I  H  K  I  R  K  Q

 451 TTTTTTCATGCTATAATGCGACAGGAGATAGGCTGGTTTGATGTG
 151  F  F  H  A  I  M  R  Q  E  I  G  W  F  D  V

 496 CACGATGTTGGGGAGCTTAACACCCGACTTACAGATGATGTCTCC
 166  H  D  V  G  E  L  N  T  R  L  T  D  D  V  S

 541 AAGATTAATGAAGGAATTGGTGACAAAATTGGAATGTTCTTTCAG
 181  K  I  N  E  G  I  G  D  K  I  G  M  F  F  Q

 586 TCAATGGCAACATTTTTCACTGGGTTTATAGTAGGATTTACACGT
 196  S  M  A  T  F  F  T  G  F  I  V  G  F  T  R

 631 GGTTGGAAGCTAACCCTTGTGATTTTGGCCATCAGTCCTGTTCTT
 211  G  W  K  L  T  L  V  I  L  A  I  S  P  V  L

 676 GGACTGTCAGCTGCTGTCTGGGCAAAGATACTATCTTCATTTACT
 226  G  L  S  A  A  V  W  A  K  I  L  S  S  F  T

 721 GATAAAGAACTCTTAGCGTATGCAAAAGCTGGAGCAGTAGCTGAA
 241  D  K  E  L  L  A  Y  A  K  A  G  A  V  A  E

 766 GAGGTCTTGGCAGCAATTAGAACTGTGATTGCATTTGGAGGACAA
 256  E  V  L  A  A  I  R  T  V  I  A  F  G  G  Q

 811 AAGAAAGAACTTGAAAGGTACAACAAAAATTTAGAAGAAGCTAAA
 271  K  K  E  L  E  R  Y  N  K  N  L  E  E  A  K

 856 AGAATTGGGATAAAGAAAGCTATTACAGCCAATATTTCTATAGGT
 286  R  I  G  I  K  K  A  I  T  A  N  I  S  I  G

 901 GCTGCTTTCCTGCTGATCTATGCATCTTATGCTCTGGCCTTCTGG
 301  A  A  F  L  L  I  Y  A  S  Y  A  L  A  F  W

 946 TATGGGACCACCTTGGTCCTCTCAGGGGAATATTCTATTGGACAA
 316  Y  G  T  T  L  V  L  S  G  E  Y  S  I  G  Q

 991 GTACTCACTGTATTCTTTTCTGTATTAATTGGGGCTTTTAGTGTT
 331  V  L  T  V  F  F  S  V  L  I  G  A  F  S  V

1036 GGACAGGCATCTCCAAGCATTGAAGCATTTGCAAATGCAAGAGGA
 346  G  Q  A  S  P  S  I  E  A  F  A  N  A  R  G

1081 GCAGCTTATGAAATCTTCAAGATAATTGATAATAAGCCAAGTATT
 361  A  A  Y  E  I  F  K  I  I  D  N  K  P  S  I

1126 GACAGCTATTCGAAGAGTGGGCACAAACCAGATAATATTAAGGGA
 376  D  S  Y  S  K  S  G  H  K  P  D  N  I  K  G

1171 AATTTGGAATTCAGAAATGTTCACTTCAGTTACCCATCTCGAAAA
 391  N  L  E  F  R  N  V  H  F  S  Y  P  S  R  K

   rs1128503 (1236 T>C) C
1216 GAAGTTAAGATCTTGAAGGGTCTGAACCTGAAGGTGCAGAGTGGG
 406  E  V  K  I  L  K  G  L  N  L  K  V  Q  S  G

1261 CAGACGGTGGCCCTGGTTGGAAACAGTGGCTGTGGGAAGAGCACA
 421  Q  T  V  A  L  V  G  N  S  G  C  G  K  S  T

1306 ACAGTCCAGCTGATGCAGAGGCTCTATGACCCCACAGAGGGGATG
 436  T  V  Q  L  M  Q  R  L  Y  D  P  T  E  G  M

1351 GTCAGTGTTGATGGACAGGATATTAGGACCATAAATGTAAGGTTT
 451  V  S  V  D  G  Q  D  I  R  T  I  N  V  R  F

1396 CTACGGGAAATCATTGGTGTGGTGAGTCAGGAACCTGTATTGTTT
 466  L  R  E  I  I  G  V  V  S  Q  E  P  V  L  F

1441 GCCACCACGATAGCTGAAAACATTCGCTATGGCCGTGAAAATGTC
 481  A  T  T  I  A  E  N  I  R  Y  G  R  E  N  V

1486 ACCATGGATGAGATTGAGAAAGCTGTCAAGGAAGCCAATGCCTAT
 496  T  M  D  E  I  E  K  A  V  K  E  A  N  A  Y

1531 GACTTTATCATGAAACTGCCTCATAAATTTGACACCCTGGTTGGA
 511  D  F  I  M  K  L  P  H  K  F  D  T  L  V  G

1576 GAGAGAGGGGCCCAGTTGAGTGGTGGGCAGAAGCAGAGGATCGCC
 526  E  R  G  A  Q  L  S  G  G  Q  K  Q  R  I  A

1621 ATTGCACGTGCCCTGGTTCGCAACCCCAAGATCCTCCTGCTGGAT
 541  I  A  R  A  L  V  R  N  P  K  I  L  L  L  D

1666 GAGGCCACGTCAGCCTTGGACACAGAAAGCGAAGCAGTGGTTCAG
 556  E  A  T  S  A  L  D  T  E  S  E  A  V  V  Q

1711 GTGGCTCTGGATAAGGCCAGAAAAGGTCGGACCACCATTGTGATA
 571  V  A  L  D  K  A  R  K  G  R  T  T  I  V  I

1756 GCTCATCGTTTGTCTACAGTTCGTAATGCTGACGTCATCGCTGGT
 586  A  H  R  L  S  T  V  R  N  A  D  V  I  A  G

1801 TTCGATGATGGAGTCATTGTGGAGAAAGGAAATCATGATGAACTC
 601  F  D  D  G  V  I  V  E  K  G  N  H  D  E  L

1846 ATGAAAGAGAAAGGCATTTACTTCAAACTTGTCACAATGCAGACA
 616  M  K  E  K  G  I  Y  F  K  L  V  T  M  Q  T

1891 GCAGGAAATGAAGTTGAATTAGAAAATGCAGCTGATGAATCCAAA
 631  A  G  N  E  V  E  L  E  N  A  A  D  E  S  K

1936 AGTGAAATTGATGCCTTGGAAATGTCTTCAAATGATTCAAGATCC
 646  S  E  I  D  A  L  E  M  S  S  N  D  S  R  S

1981 AGTCTAATAAGAAAAAGATCAACTCGTAGGAGTGTCCGTGGATCA
 661  S  L  I  R  K  R  S  T  R  R  S  V  R  G  S

2026 CAAGCCCAAGACAGAAAGCTTAGTACCAAAGAGGCTCTGGATGAA
 676  Q  A  Q  D  R  K  L  S  T  K  E  A  L  D  E

2071 AGTATACCTCCAGTTTCCTTTTGGAGGATTATGAAGCTAAATTTA
 691  S  I  P  P  V  S  F  W  R  I  M  K  L  N  L

2116 ACTGAATGGCCTTATTTTGTTGTTGGTGTATTTTGTGCCATTATA
 706  T  E  W  P  Y  F  V  V  G  V  F  C  A  I  I

2161 AATGGAGGCCTGCAACCAGCATTTGCAATAATATTTTCAAAGATT
 721  N  G  G  L  Q  P  A  F  A  I  I  F  S  K  I

2206 ATAGGGGTTTTTACAAGAATTGATGATCCTGAAACAAAACGACAG
 736  I  G  V  F  T  R  I  D  D  P  E  T  K  R  Q

2251 AATAGTAACTTGTTTTCACTATTGTTTCTAGCCCTTGGAATTATT
 751  N  S  N  L  F  S  L  L  F  L  A  L  G  I  I

2296 TCTTTTATTACATTTTTCCTTCAGGGTTTCACATTTGGCAAAGCT
 766  S  F  I  T  F  F  L  Q  G  F  T  F  G  K  A

2341 GGAGAGATCCTCACCAAGCGGCTCCGATACATGGTTTTCCGATCC
 781  G  E  I  L  T  K  R  L  R  Y  M  V  F  R  S

2386 ATGCTCAGACAGGATGTGAGTTGGTTTGATGACCCTAAAAACACC
 796  M  L  R  Q  D  V  S  W  F  D  D  P  K  N  T

2431 ACTGGAGCATTGACTACCAGGCTCGCCAATGATGCTGCTCAAGTT
 811  T  G  A  L  T  T  R  L  A  N  D  A  A  Q  V

2476 AAAGGGGCTATAGGTTCCAGGCTTGCTGTAATTACCCAGAATATA
 826  K  G  A  I  G  S  R  L  A  V  I  T  Q  N  I

2521 GCAAATCTTGGGACAGGAATAATTATATCCTTCATCTATGGTTGG
 841  A  N  L  G  T  G  I  I  I  S  F  I  Y  G  W

2566 CAACTAACACTGTTACTCTTAGCAATTGTACCCATCATTGCAATA
 856  Q  L  T  L  L  L  L  A  I  V  P  I  I  A  I

2611 GCAGGAGTTGTTGAAATGAAAATGTTGTCTGGACAAGCACTGAAA
 871  A  G  V  V  E  M  K  M  L  S  G  Q  A  L  K

     rs2032582 (2677 T>G) G
2656 GATAAGAAAGAACTAGAAGGTTCTGGGAAGATCGCTACTGAAGCA
 886  D  K  K  E  L  E  G  S  G  K  I  A  T  E  A
```

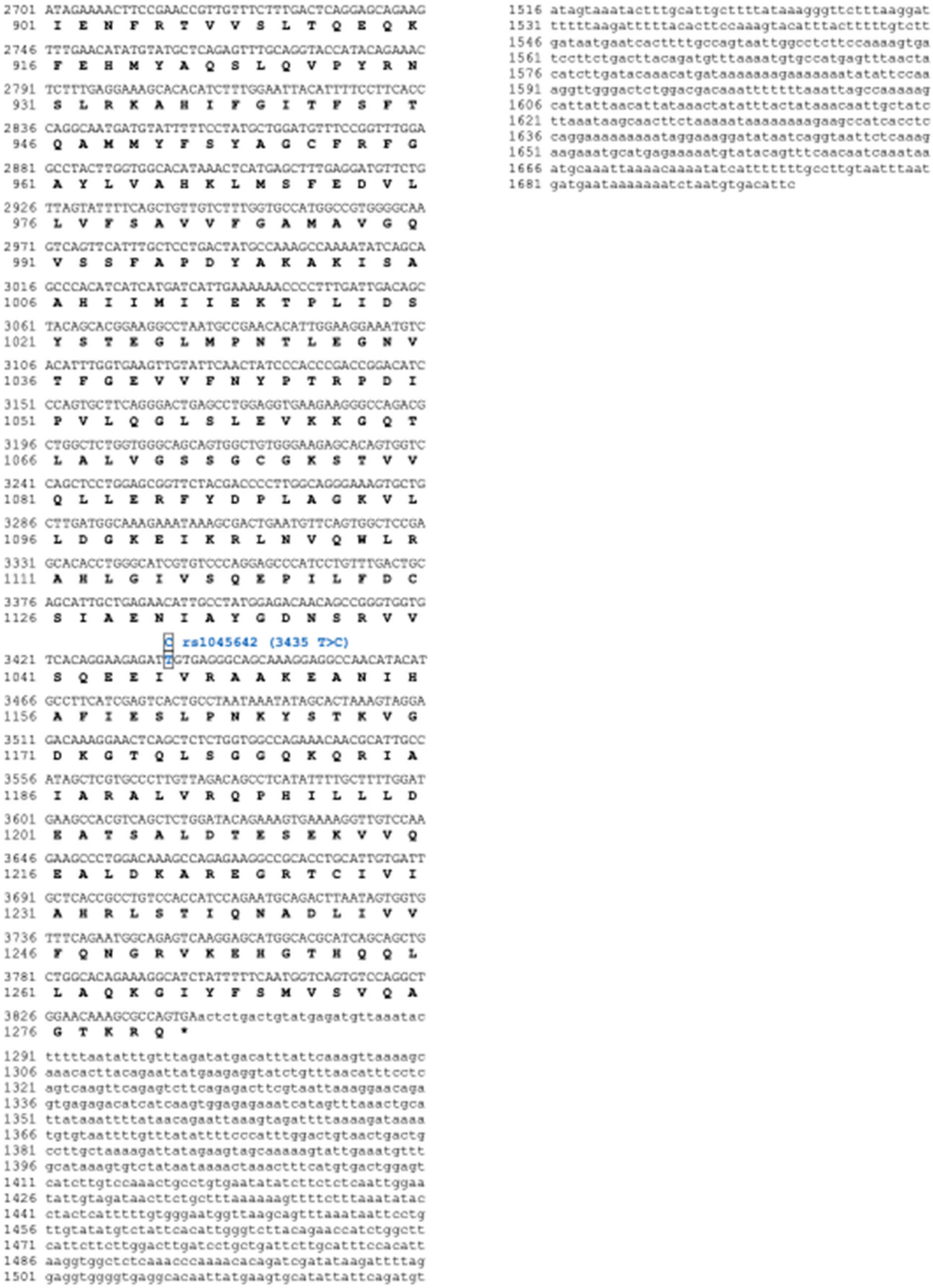

Figure S1: Human MDR-1. Homo sapiens MDR-1 nucleotide (GenBank Acc. No. NM_001348946.2) and amino acid (GenBank Acc. No. NP_001335875.1) sequence. Numbers on the left refer to the nucleotide (upper row) and amino acid (lower row) positions. Nucleotides are numbered, starting from the first ATG initiation codon within a strong Kozak

consensus sequence. * indicates the stop codon. The polymorphisms studied in this paper are marked in blue. rs1128503: the MDR-1 1236T>C is a synonymous mutation and it does not result in an amino acid change (G412G). rs2032582: the MDR-1 2677T>G is a nonsynonymous mutation resulting in a Ser-to-Ala amino acid change (Ser893Ala). rs1045642: the MDR-1 3435T>C is a synonymous mutation and it does not result in an amino acid change (Ile1145Ile).

A

```
#
#
#  Percent Identity  Matrix - created by Clustal2.1
#
#
```

1: NP_035205.1	100.00	80.35	79.97	65.98	67.35	67.54	69.32	70.51
2: NP_001335875.1	80.35	100.00	90.77	70.26	71.35	69.77	72.80	74.94
3: NP_001003215.2	79.97	90.77	100.00	69.65	71.37	69.79	72.53	74.82
4: XP_043923456.1	65.98	70.26	69.65	100.00	71.60	69.18	69.93	72.63
5: XP_030055620.1	67.35	71.35	71.37	71.60	100.00	69.94	71.43	73.06
6: XP_033021293.1	67.54	69.77	69.79	69.18	69.94	100.00	72.95	76.87
7: XP_004186266.4	69.32	72.80	72.53	69.93	71.43	72.95	100.00	78.96
8: XP_014427174.1	70.51	74.94	74.82	72.63	73.06	76.87	78.96	100.00

B

```
[…]

Homo_sapiens|primates|NP_001335875.1                 EFRNVHFSYPSRKEVKILKGLNLKVQSGQTVALVGNSGCGKSTTVQLMQRLYDPTEGMVS    452
Mus_musculus|rodents|NP_035205.1                     EFKNVHFNYPSRSEVQILKGLNLKVKSGQTVALVGNSGCGKSTTVQLMQRLYDPLEGVVS    451
Canis_lupus_familiaris|carnivores|NP_001003215.2     EFKNVHFSYPSRKEVKILKGLNLKVQSGQTVALVGNSGCGKSTTVQLMQRLYDPTDGMVC    454
Taeniopygia_guttata|birds|XP_004186266.4             ELKNVYFNYPSRPDVEILKGLNLKINSGQTVALVGGSGCGKSTTVQLIQRFYDPKEGTIT    460
Lacerta_agilis|lizards&snakes|XP_033021293.1         AFQNVHFKYPSRPDVQVLKGLNLKVNSGQTVALVGSSGCGKSTTVQLIQRFYDPLEGMIT    462
Pelodiscus_sinensis|turtles|XP_014427174.1           EFHNVFFNYPSRQDVKILKGLNLKVNCGQTVALVGSSGCGKSTTVQLIQRFYDPEKGMIT    455
Microcaecilia_unicolor|caecilians|XP_030055620.1     EFRNIHFSYPSRAEVSVLKGLNLKIQSGQTVALVGSSGCGKSTTVQLIQRFYDPESGEIT    466
Protopterus_annectens|lungfishes|XP_043923456.1      EFKNIQFTYPSRPDVKILKGLNLRINSGQTVALVGSSGCGKSTTVQLLQRFYDPEEGMVT    448
                                                       ::*: *.**** :*.:*****:::.********.***********:**:*** .* :

[…]

Homo_sapiens|primates|NP_001335875.1                 LLLAIVPIIAIAGVVEMKMLSGQALKDKKELEGSGKIATEAIENFRTVVSLTQEQKFEHM    919
Mus_musculus|rodents|NP_035205.1                     LLVVIIPLIVLGGIIEMKLLSGQALKDKKQLEISGKIATEAIENFRTIVSLTREQKFETM    917
Canis_lupus_familiaris|carnivores|NP_001003215.2     LLLAIVPIIAIAGVVEMKMLSGQALKDKKELEGAGKIATEAIENFRTVVSLTREQKFEYM    921
Taeniopygia_guttata|birds|XP_004186266.4             LLLAVVPIIAVAGMIEMKMLAGHAKKDKRELEAAGKIATEAIENIRTVVSLTLERKFELM    938
Lacerta_agilis|lizards&snakes|XP_033021293.1         VILAVVPLVAIGGLIEMRMLAGHARKDKKELEGAGKIATEAIGNIRTVVSLTQEIRFEQM    927
Pelodiscus_sinensis|turtles|XP_014427174.1           LILAVVPVIAIAGIIEMKMLSGHAKKDKKELEAAGKIATEAIENIRTVVTLTKERKFEYM    915
Microcaecilia_unicolor|caecilians|XP_030055620.1     LILAIVPFIAISGVIQMKMLAGHAKKDKKELEVAGKIASDAVENIRTVVSLTREKKFESL    933
Protopterus_annectens|lungfishes|XP_043923456.1      LILGIVPVLAISGLIQMRMLSGHAKKDKDELETAGKIATEAVENIRTVVSLTRERKFEDM    910
                                                     ::: ::*.:.:.*:::*::*:*:* *** :** :****::*: *:**:*:** * :** :

[…]

Homo_sapiens|primates|NP_001335875.1                 EIKRLNVQWLRAHLGIVSQEPILFDCSIAENIAYGDNSRVVSQEEIVRAAKEANIHAFIE    1159
Mus_musculus|rodents|NP_035205.1                     EIKQLNVQWLRAHLGIVSQEPILFDCSIAENIAYGDNSRAVSHEEIVRAAKEANIHQFID    1157
Canis_lupus_familiaris|carnivores|NP_001003215.2     EIKHLNVQWLRAHLGIVSQEPILFDCSIAENIAYGDNSRVVSHEEIMQAAKEANIHHFIE    1161
Taeniopygia_guttata|birds|XP_004186266.4             NAKALNIQWLRAQIGIVSQEPILFDCTIAENIAYGDNSREVSFEEIVSAAKQANIHSFID    1178
Lacerta_agilis|lizards&snakes|XP_033021293.1         GATQLNVQWLRSQIGIVSQEPVLFDCTIAENIAYGDNSRKVSFDEIVEVAKAANIHAFID    1167
Pelodiscus_sinensis|turtles|XP_014427174.1           HAKALNIQWLRAQIGIVSQEPILFDCSIAENIAYGDNNREVSHEEIINAAKAANIHSFID    1155
Microcaecilia_unicolor|caecilians|XP_030055620.1     DVKNLNIQWLRAQMGIVSQEPILFDCSIHENIAYGDNSREVSEDQIVSAAKAANIHSFID    1173
Protopterus_annectens|lungfishes|XP_043923456.1      DIKQLNIEWIRAQMGIVSQEPVLFDCSIADNIKYGDNTRQVAQEEVIAAAKAANIHSFIE    1150
                                                        . **::*:*:::*******:****:* :** ****.* *: :::: .** **** **:
```

Figure S2: MDR-1 sequence conservation **(A)** Overall percent identity across Dipnotetrapodomorpha MDR-1 amino acid sequences. **(B)** Multiple sequence alignment of human (*Homo sapiens*), house mouse (*Mus musculus*), dog (*Canis lupus familiaris*), zebra finch (*Taeniopygia guttata*), sand lizard (*Lacerta agilis*), Chinese soft-shelled turtle (*Pelodiscus sinensis*), *Microcaecilia unicolor* and West African lungfish (*Protopterus annectens*) MDR-1 amino acid sequence regions surrounding the three polymorphisms (centered on the amino acids boxed and typed in blue) studied in this paper (for details see Figure S1). Multiple sequence alignment and percent identity matrix were obtained by Clustal Omega at https://www.ebi.ac.uk/jdispatcher/msa/clustalo.

A

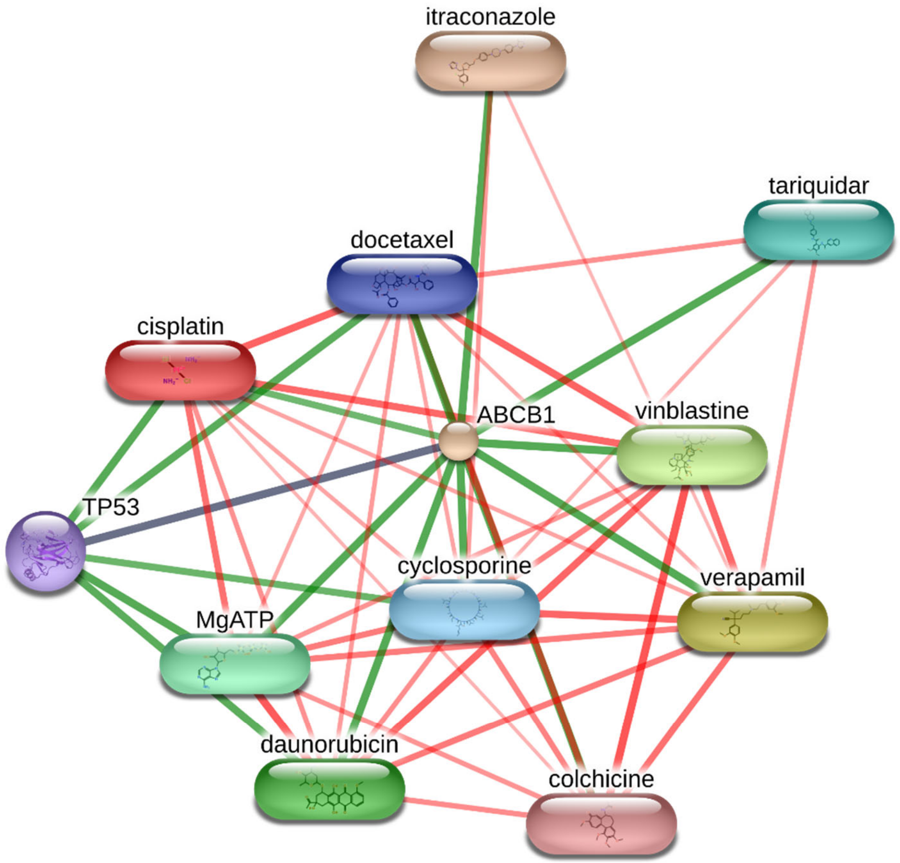

Input 1 (cisplatinum; cells)

cisplatin	Cisplatin, cisplatinum or cis-diamminedichloroplatinum(II) (CDDP) is a platinum-based chemotherapy drug used to treat various types of cancers, including sarcomas, some carcinomas (e.g. small cell lung cancer, and ovarian cancer), lymphomas and germ cell tumors. It was the first member of its class, which now also includes carboplatin and oxaliplatin (298.0 g/mol)
ABCB1	ATP-binding cassette, sub-family B (MDR/TAP), member 1; Energy-dependent efflux pump responsible for decreased drug accumulation in multidrug-resistant cells (1280 aa)

Predicted functional partners

		Score
verapamil	Verapamil (INN) (sold under various trade names) is an L-type calcium channel blocker of the phenylalkylamine class. It has been used in the treatment of hypertension, angina pectoris, cardiac arrhythmia, and most recently, cluster headaches. It is also an effective preventive medication for migraine. Verapamil has also been used as a vasodilator during cryopreservation of blood vessels. It is a class-IV antiarrhythmic, more effective than digoxin in controlling ventricular rate and was approved by the U.S. Food and Drug Administration (FDA) in March 1982 (454.6 g/mol)	0.997
vinblastine	Antitumor alkaloid isolated from *Vinca rosea* (811.0 g/mol)	0.996
daunorubicin	A very toxic anthracycline aminoglycoside antineoplastic isolated from *Streptomyces peucetius* and others, used in treatment of leukemia and other neoplasms (527.5 g/mol)	0.995
MgATP	An adenine nucleotide containing three phosphate groups esterified to the sugar moiety. In addition to its crucial roles in metabolism adenosine triphosphate is a neurotransmitter (507.2 g/mol)	0.994
tariquidar	Tariquidar (INN/USAN) is a P-glycoprotein inhibitor undergoing research as an adjuvant against multidrug resistance in cancer (646.7 g/mol)	0.993
cyclosporine	A cyclic undecapeptide from an extract of soil fungi. It is a powerful immunosupressant with a specific action on T-lymphocytes. It is used for the prophylaxis of graft rejection in organ and tissue transplantation. Cyclosporine is produced as a metabolite by the fungus species *Cordyceps militaris* (1202.6 g/mol)	0.993
docetaxel	Docetaxel is a clinically well established anti-mitotic chemotherapy medication used mainly for the treatment of breast, ovarian, and non-small cell lung cancer. Docetaxel binds to microtubules reversibly with high affinity and has a maximum stoichiometry of one mole docetaxel per mole tubulin in microtubules (807.9 g/mol)	0.991
TP53	tumor protein p53; Acts as a tumor suppressor in many tumor types; induces growth arrest or apoptosis depending on the physiological circumstances and cell type. Involved in cell cycle regulation as a trans-activator that acts to negatively regulate cell division by controlling a set of genes required for this process. One of the activated genes is an inhibitor of cyclin-dependent kinases. Apoptosis induction seems to be mediated either by stimulation of BAX and FAS antigen expression, or by repression of Bcl-2 expression (By similarity) (393 aa)	0.990
colchicine	A major alkaloid from *Colchicum autumnale* L. and found also in other *Colchicum* species. Its primary therapeutic use is in the treatment of gout, but it has been used also in the therapy of familial Mediterranean fever (periodic disease) (399.4 g/mol)	0.990

	itraconazole	One of the triazole antifungal agents that inhibits cytochrome P-450-dependent enzymes resulting in impairment of ergosterol synthesis. It has been used against histoplasmosis, blastomycosis, cryptococcal meningitis & aspergillosis (705.6 g/mol)	0.988

B

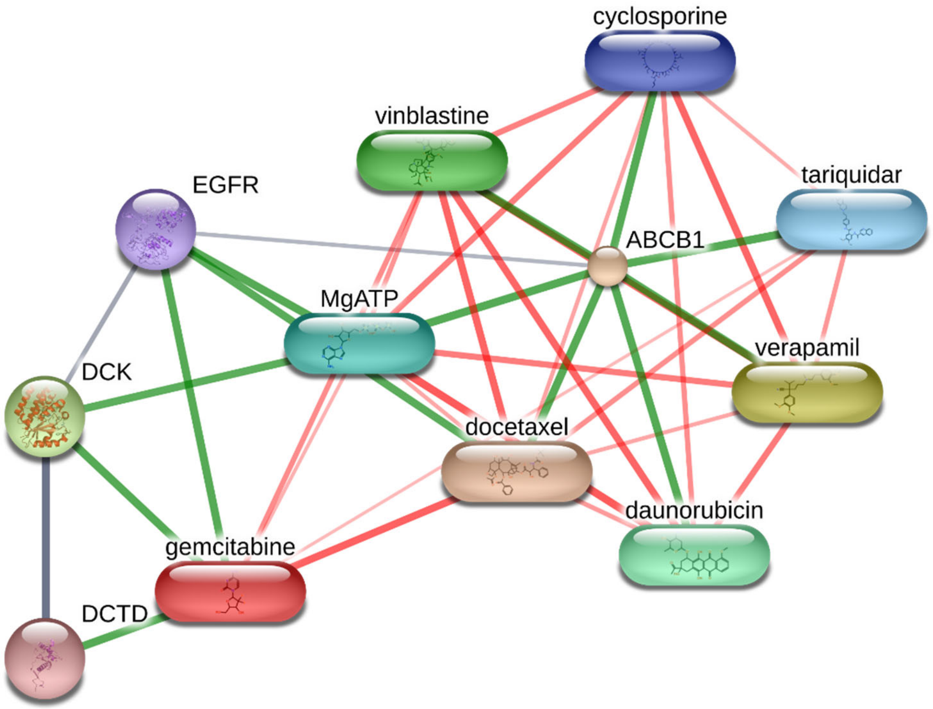

Input 2 (gemcitabine; cells)

	gemcitabine	Gemcitabine is a nucleoside analog used as chemotherapy. It is marketed as Gemzar by Eli Lilly and Company. As with fluorouracil and other analogues of pyrimidines, the drug replaces one of the building blocks of nucleic acids, in this case cytidine, during DNA replication. The process arrests tumor growth, as new nucleosides cannot be attached to the “faulty” nucleoside, resulting in apoptosis (cellular “suicide”) (263.2 g/mol)
	ABCB1	ATP-binding cassette, sub-family B (MDR/TAP), member 1; Energy-dependent efflux pump responsible for decreased drug accumulation in multidrug-resistant cells (1280 aa)

Predicted functional partners

		Score
verapamil	Verapamil (INN) (sold under various trade names) is an L-type calcium channel blocker of the phenylalkylamine class. It has been used in the treatment of hypertension, angina pectoris, cardiac arrhythmia, and most recently, cluster headaches. It is also an effective preventive medication for migraine. Verapamil has also been used as a vasodilator during cryopreservation of blood vessels. It is a class-IV antiarrhythmic, more effective than digoxin in controlling ventricular rate and was approved by the U.S. Food and Drug Administration (FDA) in March 1982 (454.6 g/mol)	0.997
DCK	deoxycytidine kinase; Required for the phosphorylation of the deoxyribonucleosides deoxycytidine (dC), deoxyguanosine (dG) and deoxyadenosine (dA). Has broad substrate specificity, and does not display selectivity based on the chirality of the substrate. It is also an essential enzyme for the phosphorylation of numerous nucleoside analogs widely employed as antiviral and chemotherapeutic agents (260 aa)	0.997
vinblastine	Antitumor alkaloid isolated from *Vinca rosea* (811.0 g/mol)	0.996
daunorubicin	A very toxic anthracycline aminoglycoside antineoplastic isolated from *Streptomyces peucetius* and others, used in treatment of leukemia and other neoplasms (527.5 g/mol)	0.995
MgATP	An adenine nucleotide containing three phosphate groups esterified to the sugar moiety. In addition to its crucial roles in metabolism adenosine triphosphate is a neurotransmitter (507.2 g/mol)	0.994
tariquidar	Tariquidar (INN/USAN) is a P-glycoprotein inhibitor undergoing research as an adjuvant against multidrug resistance in cancer (646.7 g/mol)	0.993
cyclosporine	A cyclic undecapeptide from an extract of soil fungi. It is a powerful immunosupressant with a specific action on T-lymphocytes. It is used for the prophylaxis of graft rejection in organ and tissue transplantation. Cyclosporine is produced as a metabolite by the fungus species *Cordyceps militaris* (1202.6 g/mol)	0.993
EGFR	epidermal growth factor receptor (1210 aa)	0.991
DCTD	dCMP deaminase; Supplies the nucleotide substrate for thymidylate synthetase (189 aa)	0.991
docetaxel	Docetaxel is a clinically well established anti-mitotic chemotherapy medication used mainly for the treatment of breast, ovarian, and non-small cell lung cancer. Docetaxel binds to microtubules reversibly with high affinity and has a maximum stoichiometry of one mole docetaxel per mole tubulin in microtubules (807.9 g/mol)	0.991

Figure S3. Protein-chemical interactions involving ABCB1, cisplatin or gemcitabine, and predicted functional partners (confidence view) as obtained by STITCH, a database of protein-chemical interactions that integrates various sources of experimental and manually curated evidence with text-mining information and interaction predictions (http://stitch.embl.de/ accessed on 25 July 2024). Stronger associations are represented by thicker lines. Protein-protein interactions are shown in grey, chemical-protein interactions in green, and interactions between chemicals in red. Analysis was conducted using *Homo sapiens* as reference species and 2 different inputs cell-based (input 1: cisplatin; input 2: gemcitabine)

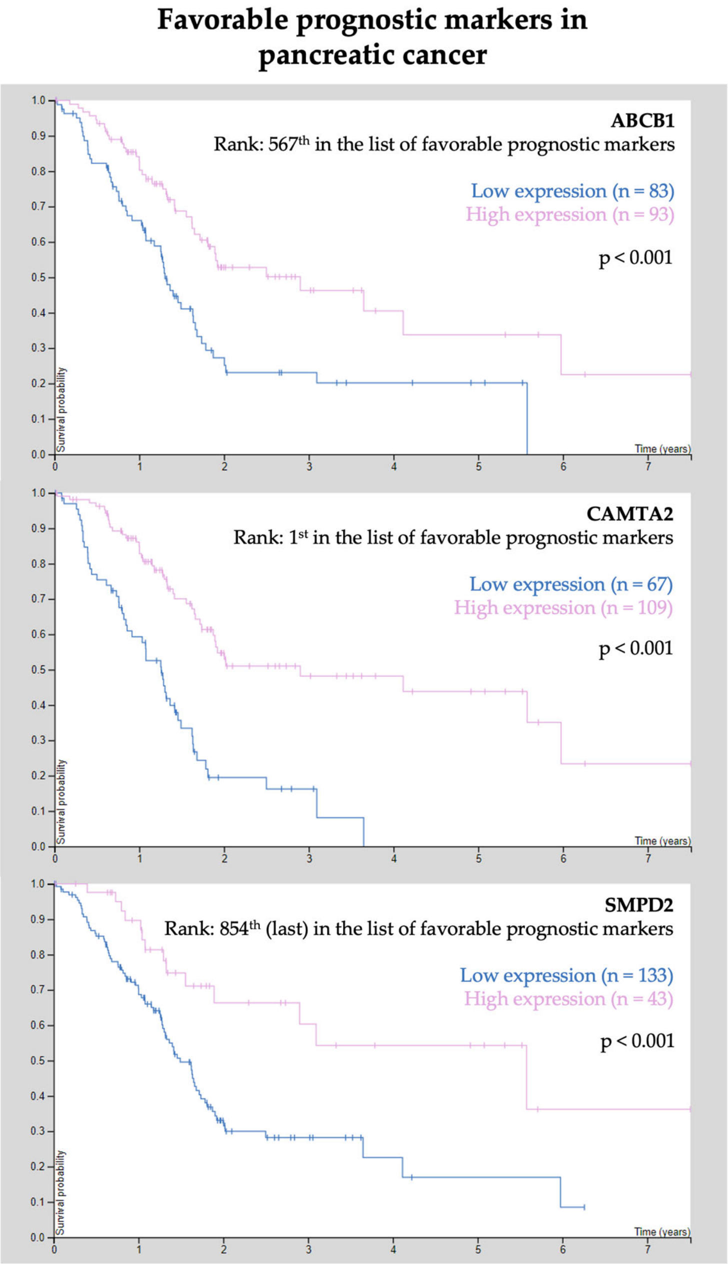

Figure S4. Kaplan-Meier Survival probability (1.0) (Y-axis) *vs.* Time (years) (X-axis) plot. The pancreatic cancer proteome was explored (query: July 25, 2024) in 'The HumanProtein Atlas' using TCGA transcriptomics data and antibody-based protein data (https://www.proteinatlas.org/ENSG00000075142-SRI/pathology/pancreatic+cancer). In the dataset, 1522 genes are suggested as prognostic based on transcriptomics data from 176 patients; 854 genes are associated with favorable prognosis. ABCB1 is compared to the first (CAMTA) and the last (SMPD2) favorable prognostic marker in pancreatic cancer. Abbreviations: ABCB1, ATP binding cassette subfamily B member 1; CAMTA2, calmodulin binding transcription activator 2; SMPD2, sphingomyelin phosphodiesterase 2.

Table S1: Primers for PCR analysis of *MDR-1* gene

Exon	Primer	Sequence (5'->3')	Length	Tm	Ta (°C)
6	Forward primer	TACCCTACACTCAAAACAGGCT	22	59.02	56
	Reverse primer	TTCCGTAGGGTGAGAGCAGG	20	60.97	
12	Forward primer	ATCCTGTGTCTGTGAATTGCCT	22	59.96	56
	Reverse primer	ATGTGACTGCTGATCACCGC	20	60.74	
14	Forward primer	TTGTGAGACTTGGGCTGTGTAT	22	59.63	53
	Reverse primer	GGTTGGTTTGAACTAAGCCTC	21	57.08	
16	Forward primer	GCTTTCATTGGTTAACACACAGC	23	59.26	55
	Reverse primer	TAGCCCTAGCCCACCAAACT	20	60.55	
21	Forward primer	GTCTCATGAAGGTGAGTTTTCAG	23	57.40	53
	Reverse primer	GAGCATAGTAAGCAGTAGGGAG	22	57.49	
26	Forward primer	ATTCAAAGTGTGCTGGTCCTG	21	58.77	54.5
	Reverse primer	GCTCCCAGGCTGTTTATTTGA	21	58.55	

Table S2: Polymorphisms in the *MDR-1 gene* reported in the literature

Exon	Polymophisms	Source
Exon 6	554 G>T	https://www.ncbi.nlm.nih.gov/variation/view/
Exon 12	1236 T>C	https://www.ncbi.nlm.nih.gov/variation/view/
Exon 14	1662 G>C	https://www.ncbi.nlm.nih.gov/variation/view/
	1662 G>A	https://www.ncbi.nlm.nih.gov/variation/view/
Exon 16	1985 T>G	https://www.ncbi.nlm.nih.gov/variation/view/
	2005 C>T	https://www.ncbi.nlm.nih.gov/variation/view/
	2005 C>A	https://www.ncbi.nlm.nih.gov/variation/view/
Exon 21	2677 T>G	https://www.ncbi.nlm.nih.gov/variation/view/
	2677 T>A	https://www.ncbi.nlm.nih.gov/variation/view/
Exon 26	3320 A>C	https://www.ncbi.nlm.nih.gov/variation/view/
	3322 T>C	https://www.ncbi.nlm.nih.gov/variation/view/
	3289 G>T	https://hive.biochemistry.gwu.edu/biomuta/proteinview/P08183
	3412 C>T	https://hive.biochemistry.gwu.edu/biomuta/proteinview/P08183
	3413 G>T	https://hive.biochemistry.gwu.edu/biomuta/proteinview/P08183
	3421 T>A	https://www.ncbi.nlm.nih.gov/variation/view/
	3435 T>G	https://www.ncbi.nlm.nih.gov/variation/view/
	3435 T>C	https://www.ncbi.nlm.nih.gov/variation/view/
	3435 T>A	https://www.ncbi.nlm.nih.gov/variation/view/

Disclaimer/Publisher's Note: The statements, opinions and data contained in all publications are solely those of the individual author(s) and contributor(s) and not of MDPI and/or the editor(s). MDPI and/or the editor(s) disclaim responsibility for any injury to people or property resulting from any ideas, methods, instructions or products referred to in the content.
